# Paediatric emergency department utilisation rates and maternal migration status in the Born in Bradford cohort: A cross-sectional study

**DOI:** 10.1371/journal.pmed.1003043

**Published:** 2020-03-03

**Authors:** Sarah H. Credé, Suzanne Mason, Elizabeth Such, Richard M. Jacques

**Affiliations:** School of Health and Related Research, University of Sheffield, Sheffield, United Kingdom; International Organization for Migration, SRI LANKA

## Abstract

**Background:**

Globally, international migration is increasing. Population growth, along with other demographic changes, may be expected to put new pressures on healthcare systems. Some studies across Europe suggest that emergency departments (EDs) are used more, and differently, by migrants compared to non-migrant populations, which may be a result of unfamiliarity with the healthcare systems and difficulties accessing primary healthcare. However, little evidence exists to understand how migrant parents, who are typically young and of childbearing age, utilise EDs for their children. This study aimed to examine the association between paediatric ED utilisation in the first 5 years of life and maternal migration status in the Born in Bradford (BiB) cohort study.

**Methods and findings:**

We analysed linked data from the BiB study—an ongoing, multi-ethnic prospective birth cohort study in Bradford. Bradford is a large, ethnically diverse city in the north of England. In 2017, more than a third of births in Bradford were to mothers who were born outside the UK. Between March 2007 and December 2010, pregnant women were recruited to BiB during routine antenatal care, and the children born to these mothers have been, and continue to be, followed over time to assess how social, genetic, environmental, and behavioural factors impact on health from childhood to adulthood. Data analysed in this study included baseline questionnaire data from BiB mothers, and Bradford Royal Infirmary ED episode data for their children. Main outcomes were likelihood of paediatric ED use (no visits versus at least 1 ED visit in the first 5 years of life) and ED utilisation rates (number and frequency of ED visits) for children who have accessed the ED. The main explanatory variable was mother’s migrant status (foreign-born versus UK/Irish-born). Multivariable analyses (logistic and zero-truncated negative binomial regression) were conducted adjusting for socio-demographic and socio-economic factors. The final dataset included 10,168 children born between April 2007 and June 2011, of whom 35.6% were born to migrant mothers. Foreign-born mothers originated from South Asia (28.6%), Europe/Central Asia (3.2%), Africa (2.1%), East Asia/Pacific (1.1%), and the Middle East (0.6%). At recruitment the mothers ranged in age from 15 to 49 years old. Overall, 3,104 (30.5%) children had at least 1 ED visit in the first 5 years of life, with the highest proportion of visits being in the first year of life (36.7%). The proportion of children who visited the ED at least once was lower for children of migrant mothers as compared to children of non-migrant mothers (29.4% versus 31.2%). Children of migrant mothers were found to be less likely to visit the ED (odds ratio 0.88 [95% CI 0.80 to 0.97], *p* = 0.012). However, among children who visited the ED, the utilisation rate was significantly higher for children of migrant mothers (incidence rate ratio [IRR] 1.19 [95% CI 1.01 to 1.40], *p* = 0.040). Utilisation rates were higher for children born to mothers from Europe (IRR 1.71 [95% CI 1.07 to 2.71], *p* = 0.024) and established migrants (≥5 years living in UK) (IRR 1.24 [95% CI 1.02 to 1.51], *p* = 0.032) compared to UK/Irish-born mothers. Important limitations include being unable to measure children’s underlying health status and the urgency of ED attendance, as well as the analysis being limited by missing data.

**Conclusions:**

In this study we observed that there is no higher likelihood of first paediatric ED attendance in the first 5 years of life for children in the BiB cohort for migrant mothers. However, among ED users, children of migrant mothers attend the service more frequently than children of UK/Irish-born mothers. Our findings show that patterns of ED utilisation differ by mother’s region of origin and time since arrival in the UK.

## Abstract 2

In a cross-sectional study, Sarah Credé and colleagues investigate paediatric Emergency Department utilization rates and maternal migration status in the Born in Bradford cohort.

## Author summary

**Why was this study done?:**

- Migrant populations are typically of childbearing age and contribute to a growing proportion of the birth rate in the UK.
- Migrant populations demonstrate different patterns of emergency department (ED) use compared to people born in country across many European states. There is limited understanding of migrant parents’ use of EDs in the UK for their children’s healthcare needs.
- We conducted a study to explore whether migrant parents use EDs in different ways to non-migrant parents for their children.

**What did the researchers do and find?:**

- We analysed patterns of ED utilisation in the first 5 years of life for 10,168 children in the Born in Bradford cohort. We compared ED use between children born to migrant (non-UK/Irish-born) mothers and UK/Irish-born mothers.
- We found that children of migrant mothers were less likely to make a first visit to the ED during their first 5 years of life, but children of migrant mothers who did use the ED were found to use it more frequently than children of UK/Irish-born mothers who used the ED.
- We also found that children of mothers with different regions of origin utilised the ED differently, and that children of migrant mothers who have been in the UK for a long time used the ED in similar patterns to children of mothers born in the UK.

**What do these findings mean?:**

- Overall, children of migrant mothers are less likely to make a first visit to the ED for their healthcare needs when compared to children of UK/Irish-born mothers. This raises the question of potential underuse, or low awareness of EDs, among some migrant groups.
- Different patterns of ED use between migrant and non-migrant parents for their children may be due to different underlying health needs in these populations, lack of understanding of the UK healthcare service, or barriers to more appropriate healthcare services.
- It is important that further research seeks to understand the reasons why some migrant families use the ED more frequently, particularly whether barriers to other healthcare exist.
- The main limitations of this study relate to the analysis being limited by missing data for some of the mothers in the cohort and being unable to measure children’s underlying health status and the urgency of their ED attendances.

## Introduction

International migration into, and within, Europe continues to increase, with migrants forming a growing proportion of the population in many countries [1]. Population growth, along with other important demographic changes, such as population ageing, can be expected to put new pressures on healthcare systems due to altered service demand [2,3]. Much discussion and political debate in the United Kingdom (UK) on migrants’ use of healthcare has centred on emergency services. It is often argued that migrants place increased pressure on already overstretched services [4]. However, these debates are often unsupported by empirical evidence as there is a paucity of detailed research on migrants’ use of emergency services.

The UK’s National Health Service (NHS) provides health services that are free for users at the point of access for any person who is ‘ordinarily resident’ in the UK [5]. These services are offered on the basis of clinical need rather than ability to pay and include primary care services, urgent and emergency care, as well as hospital services. Importantly, any person, irrespective of migrant status or time since arrival in the UK, is currently entitled to free general practitioner (GP), primary care, and emergency services [5]. Parents, or caregivers, can access urgent and emergency care for their children through their GP; via NHS 111, which provides online or telephone advice to people with urgent medical problems; by making an emergency call; by accessing a walk-in centre or minor injuries unit; or by accessing an emergency department (ED). No referral is needed to access an ED. EDs provide 24-hour urgent and emergency care, while GPs, walk-in centres, and minor injuries units have restricted opening hours, with some services having a provision for out-of-hours urgent care. In non-emergency situations, parents and caregivers are encouraged, where possible, to consult their GP, or to access other urgent care services, before using an ED.

The current demand for NHS emergency care is unprecedented. Adding to this pressure, and of concern, is the increase in ED attendances and admissions of children under 5 years over the last decade [6]. Of these attendances, 60% are non-urgent [7]. Children are often vulnerable users of emergency services, dependent on their caregivers when accessing care [6]. Migrants to the UK are typically young and of childbearing age [8] and contribute to a substantial proportion of the UK birth rate [9]. It is therefore important to understand whether differences in patterns of paediatric ED use by migrant populations, as compared to the UK-born population, may contribute to increased paediatric ED use.

In many other European countries, higher use of EDs by immigrants, compared to people born in-country, has been observed in both children and adults [10]. Evidence suggests migrants tend to access EDs for low-acuity presentations, which indicates that barriers to more appropriate primary healthcare services exist [10]. To ensure the provision of appropriate and accessible emergency services to the demographically changing population, and to manage demand for these services, it is essential to understand paediatric ED utilisation by migrant caregivers for their children [11].

This study aimed to establish whether there are differences in paediatric ED utilisation in the first 5 years of life for children born to migrant, as compared to non-migrant, mothers in the Born in Bradford (BiB) cohort.

## Methods

### Participants

We used data from the BiB cohort study [12], an ongoing birth cohort study in the city of Bradford, a city in the north of England that is ethnically diverse and has high levels of residential deprivation. Approximately 16% of the community living in Bradford is non-UK-born, and 34% of births in this area are to mothers who themselves were born outside the UK [13].

Between March 2007 and December 2010, 12,453 pregnant women were recruited to BiB during routine antenatal care. The babies born to these mothers have been, and continue to be, followed over time to assess how social, genetic, environmental, and behavioural factors impact on health from childhood into adulthood [14]. Detailed methods for the BiB study have been reported elsewhere [14]. This study is reported as per the Strengthening the Reporting of Observational Studies in Epidemiology (STROBE) guideline (S1 Checklist) [15].

Data for this study included BiB baseline questionnaire data [16] matched on an individual patient level basis—using an exact match on NHS number, surname, date of birth, and sex—to Bradford Royal Infirmary (BRI) ED attendance records [17]. The baseline questionnaire was completed by all mothers at recruitment and included socio-demographic, general health, financial, social, and environmental characteristics. Detailed migration history was collected, including country of birth data for both parents and time since mother’s arrival in the UK.

The ED attendance data contain information about all ED visits to BRI in the first 5 years of the child’s life, including the date of attendance, date of discharge, and presenting condition, coded using the International Classification of Diseases–10th revision (ICD-10) codes. BRI ED is 1 of 2 EDs that serve Bradford and the surrounding districts, with BRI ED primarily serving the population of Bradford [18].

### Ethics

The BiB cohort participants provided written informed consent for data collection and granted permission for the study to access routine medical records. Ethical approval for the data collection was granted by Bradford Research Ethics Committee (Ref 07/H1302/112).

### Outcomes of interest and other variables

The main outcome of interest was any ED use at BRI in first 5 years of life (no visits versus at least 1 ED visit). We also analysed utilisation rates, considering the number and frequency of ED visits by children who used the ED. As some children in the cohort were born to the same mother, first ED visit was defined as the first ED attendance per child.

The main explanatory variable was mother’s migrant status. A migrant mother was defined as a mother who was born outside the UK or Ireland. UK- or Irish-born mothers were considered non-migrants.

Migrants are not a homogeneous group; populations will have had different experiences in their host country and are ethnically, socio-economically, and socio-demographically diverse, which may affect health and healthcare use [19]. To gain a deeper understanding of the relationship between migration status and health, sub-group analyses were conducted by mother’s region of birth, according to World Bank regions, and by mother’s time since arrival in the UK at time of recruitment: short-term migrant (<1 year in UK), long-term migrant (living in UK ≥1 to <5 years), or established migrant (≥5 years in UK).

Health condition diagnoses were coded using the ICD-10 system and grouped according to categories. Type of discharge from the ED was categorised as either discharged from ED (0 days’ stay) or admitted to hospital (≥2 days’ length of stay). Children who were categorised as having a duration of stay of 1 day were excluded for this analysis. These children were excluded because, in the dataset, any child whose date of ED attendance was different to their discharge date was recorded as having a duration of stay ≥1 day. However, this may include children who attended late at night and who were discharged the following morning, or the following day, from the ED (duration of attendance of 1 day), although these children will not have been admitted to an in-patient ward.

The analysis was restricted to all children for whom there was complete country of birth data for the mother as well as complete data for the covariates of interest. The final cohort included 10,168 children. A comparison of the children included and those excluded from the analysis due to missing data is reported in S1 Table.

### Statistical analysis

A prospective protocol for analysis was not prepared for this study; however, all analyses were planned in advance of data analysis. Descriptive analyses were undertaken to identify the differences in socio-demographic characteristics between migrant and non-migrant mothers using Pearson chi-squared tests or Fisher’s exact tests for categorical variables, and Mann–Whitney U tests for numeric variables. To describe paediatric ED utilisation, summary statistics were calculated along with ED utilisation rates. Crude ED utilisation rates per 1,000 children per year were calculated as the ratio of the number of ED episodes recorded for each sub-group of interest divided by the number of children in that particular sub-group. This ratio was divided by the number of years of follow-up (5) and multiplied by 1,000.

Two approaches were used to analyse patterns of paediatric ED utilisation. We assessed the likelihood of any ED use in the full cohort using unadjusted and multiple logistic regression models. We then conducted zero-truncated negative binomial regression to model the count of ED visits among ED users, i.e., conditional on ever using the ED, to study the frequency of use (number of visits over the first 5 years of life). Regression coefficients from zero-truncated negative binomial regression models are interpreted as incidence rate ratios (IRRs).

Separate models were fitted for mothers’ migrant status (migrant yes/no), time since mothers’ arrival in the UK, and mothers’ region of origin. In all models, UK/Irish-born mothers were the reference population. For each multivariable model we controlled for covariates of interest including child’s sex, mother’s age, parity (no previous live births versus at least 1 previous birth), maternal education (less than A level or equivalent qualification, A level or higher qualification, or unknown highest level of education), maternal level of residential deprivation using Index of Multiple Deprivation (IMD) [20] quintiles derived from national data zones, and distance from home to BRI ED in kilometres (calculated from postcode at time of birth). These covariates were included in the multivariable analysis based on knowledge about the relationship between covariates and migrant status or a priori hypothesised relationships with the outcome of interest, and were further considered if the variable showed a significant association in univariable logistic regression analyses (*p* < 0.05). Continuous covariates were included in the model as linear terms. Zero-truncated negative binomial regression was chosen because, among users of the ED, the possibility of a count of 0 attendances is not possible and the use of negative binomial regression is inappropriate [21]. All analyses were undertaken using Stata 14 [22]. Significance was accepted at the 5% level (*p* < 0.05).

## Results

### Description of the cohort

The cohort included 10,168 children, of whom 3,620 (35.6%) were born to migrant mothers. The majority of migrant mothers were from South Asia (Table 1), of whom 88.2% were of Pakistani origin. At the time of recruitment, 58.7% of migrant mothers had been living in the UK for at least 5 years, 35.2% had been in the UK for 1 to ≤5 years, and 6.1% had lived in the UK for less than 1 year at time of study recruitment. The relative deprivation in this cohort, particularly among migrant mothers, is highlighted by the large proportion of mothers who lived in the most deprived areas of Bradford (Table 1). Migrant mothers, on average, lived closer to BRI ED, and for a greater proportion of these mothers (99.9% versus 97.9%), BRI was the closest ED to their home at the time of their child’s birth.

**Table 1:** Socio-demographic and socio-economic characteristics of mothers in the Born in Bradford (BiB) cohort.

Characteristic	Total (*N* = 10,168)	UK/Irish-born mothers (*N* = 6,548)	Migrant mothers (*N* = 3,620)	*p*-Value*
**Region of origin**				
UK/Ireland	6,548 (64.4%)			
South Asia	2,913 (28.6%)			
Europe/Central Asia	322 (3.2%)			
Africa	211 (2.1%)			
East Asia/Pacific	112 (1.1%)			
Middle East	62 (0.6%)			
**Child sex**				0.230
Male	5,157 (50.7%)	3,350 (51.2%)	1,807 (49.9%)	
Female	5,011 (49.3%)	3,198 (48.8%)	1,813 (50.1%)	
**Mother’s age at recruitment (years)**				
Median (IQR)	27 (23; 31)	26 (22; 31)	28 (24; 32)	**<0.001**
Range	15–49	15–45	15–49	
**Parity**				**<0.001**
No previous birth	4,179 (41.1%)	2,961 (45.2%)	1,218 (33.6%)	
At least 1 previous birth	5,989 (58.9%)	3,587 (54.8%)	2,402 (66.4%)	
**Maternal education**				**<0.001**
Less than A level or equivalent	5,286 (52.0%)	3,348 (51.1%)	1,938 (53.5%)	
A level equivalent or higher	4,670 (45.9%)	3,140 (48.0%)	1,530 (42.3%)	
Don’t know/foreign unknown	212 (2.1%)	60 (0.9%)	152 (4.2%)	
**Marital and cohabitation status**				**<0.001**
Married and living with partner	6,642 (65.3%)	3,383 (51.7%)	3,259 (90.0%)	
Not married, living with partner	1,838 (18.1%)	1,686 (25.7%)	152 (4.2%)	
Not living with partner	1,688 (16.6%)	1,479 (22.6%)	209 (5.8%)	
**Residential deprivation IMD quintile 2010**				**<0.001**
1 (most deprived)	6,712 (66.0%)	3,887 (59.4%)	2,825 (78.0%)	
2	1,838 (18.1%)	1,278 (19.5%)	560 (15.5%)	
3	1,141 (11.2%)	948 (14.5%)	193 (5.3%)	
4	302 (3.0%)	274 (4.2%)	28 (0.8%)	
5 (least deprived)	175 (1.7%)	161 (2.4%)	14 (0.4%)	
**Closest ED to home**				**<0.001**
Bradford Royal Infirmary	10,029 (98.6%)	6,414 (97.9%)	3,615 (99.9%)	
Other	139 (1.4%)	134 (2.1%)	5 (0.1%)	
**Distance from home to hospital (km)**				
Median (IQR)	3.61 (2.14; 4.88)	4.06 (2.56; 5.23)	2.71 (1.42; 4.24)	**<0.001**
Range	0.33–21.14	0.33–21.14	0.33–10.77	

Data are *n* (%) unless otherwise indicated. Significant *p*-values (*p* < 0.05) are bolded.

*Chi-squared analysis for categorical variables; Mann–Whitney U analysis for numeric variables.

ED, emergency department; IMD, Index of Multiple Deprivation.

### ED attendance and volume of utilisation

Overall, 3,104 (30.5%) children had at least 1 ED attendance in the first 5 years of life, with a total of 5,395 ED visits (Table 2). The proportion of children who ever visited the ED was lower for children of migrant mothers (29.4% versus 31.2%).

**Table 2:** ED visit frequency by maternal migrant status and migrant sub-group.

Outcome	Total *N* (%)	Migrant status		Mother’s region of origin					Mother’s time since arrival in the UK		
		UK/Irish-born mothers (reference)	Migrant mothers	Europe/Central Asia	East Asia/Pacific	Middle East	South Asia	Africa	Established (≥ 5 years in UK)	Long term (≥1 to <5 years in UK)	Short term (<1 year in UK)
**Study cohort *N* (%)**	10,168 (100%)	6,548 (64.4%)	3,620 (35.6%)	322 (3.2%)	112 (1.1%)	62 (0.6%)	2,913 (28.6%)	211 (2.1%)	2,125 (21.0%)	1,274 (12.5%)	221 (2.2%)
**Total ED visits (count)**	5,395 (100%)	3,406 (63.2%)	1,989 (36.8%)	179 (3.3%)	49 (0.9%)	24 (0.4%)	1,636 (30.3%)	101 (1.9%)	1,232 (22.8%)	646 (11.9%)	111 (2.1%)
**Number of ED visits per child**											
No visits	7,064 (69.5%)	4,506 (68.8%)	2,558 (70.6%)	243 (75.5%)	83 (74.1%)	49 (79.0%)	2,020 (69.3%)	163 (77.3%)	1,465 (68.9%)	930 (73.0%)	163 (73.8%)
At least 1	3,104 (30.5%)	2,042 (31.2%)	1,062 (29.4%)	79 (24.5%)	29 (25.9%)	13 (21.0%)	893 (30.7%)	48 (22.7%)	660 (31.1%)	344 (27.0%)	58 (26.2%)
Crude ED utilisation rate per 1,000 children in cohort per year*	106.12	104.03	109.89	111.18	87.50	77.42	112.32	95.73	115.95	101.41	100.45
**ED use among children who made use of the ED**											
Number of ED users^#^	3,104	2,042	1,062	79	29	13	893	48	660	344	58
Mean number of ED visits per user^#^	1.74	1.67	1.87	2.27	1.69	1.84	1.83	2.10	1.87	1.88	1.91
Crude ED utilisation rate per 1,000 children using the ED per year^	347.62	333.59	374.58	453.16	337.93	369.2	366.40	420.83	373.33	375.58	382.76

*Crude ED utilisation rate per 1,000 children in cohort per year calculated as the ratio of the number of ED episodes recorded for each sub-group of interest divided by the number of children in that particular group. This ratio was divided by the number of years of follow-up (5) and multiplied by 1,000.

^#^User is defined as a child who made at least 1 visit to the ED in the first 5 years of life.

^Crude ED utilisation rate per 1,000 children using the ED per year is calculated as the ratio of the number of ED episodes recorded for each sub-group of children using the ED divided by the number of children in that particular group. This ratio was divided by the number of years of follow-up (5) and multiplied by 1,000.

ED, emergency department.

The greatest proportion of ED attendances for this cohort of children took place in the first year of life (Table 3). Patterns of ED utilisation across weekends and weekdays were similar for both groups. Of the 5,395 ED attendances, 57.3% resulted in a hospital admission of at least 2 days. The most common reason for ED attendance, for both migrant and non-migrant children, was for respiratory conditions, with infectious diseases and injuries being the next most common reasons for presentations. For children who attended the ED, the average distance from home to hospital was 3.56 km, with migrants, on average, living closer to the BRI ED (2.58 versus 4.05 km).

**Table 3:** Details of ED attendances (*N* = 5,395).

Characteristic	Total ED visits (*N* = 5,395)	Children of UK/Irish-born mothers (*N* = 3,406)	Children of migrant mothers (*N* = 1,989)
**Child sex**			
Male	3,026 (56.1%)	1,892 (55.5%)	1,134 (57.0%)
Female	2,369 (43.9%)	1,514 (44.5%)	855 (43.0%)
**Age of child at time of attendance**			
0 to <1 year	1,978 (36.7%)	1,282 (37.7%)	696 (35.0%)
1 to <2 years	1,252 (23.2%)	808 (23.7%)	444 (22.3%)
2 to <3 years	844 (15.6%)	518 (15.2%)	326 (16.4%)
3 to <4 years	747 (13.9%)	468 (13.7%)	279 (14.0%)
4 to <5 years	574 (10.6%)	330 (9.7%)	244 (12.3%)
**Type of day**			
Weekday	3,888 (72.1%)	2,437 (71.5%)	1,451 (72.9%)
Weekend/bank holiday	1,507 (27.9%)	969 (28.5%)	538 (27.1%)
**Type of discharge from ED**			
Discharged (0 days’ admission)	1,615 (42.7%)	1,048 (44.2%)	566 (40.0%)
≥2 days’ hospital admission	2,171 (57.3%)	1,322 (55.8%)	849 (60.0%)
**Duration of admission (days) (for those duration of stay ≥ 2 days, *N* = 2,171)**			
Median (IQR)	2 (2; 4)	3 (2; 4)	3 (2; 5)
Range	2–111	2–49	2–111
**ICD-10 code for ED attendance**			
Respiratory disease	2,024 (37.5%)	1,257 (36.9%)	767 (38.6%)
Infectious disease	974 (18.1%)	619 (18.2%)	355 (17.8%)
Injury	641 (11.9%)	430 (12.6%)	211 (10.6%)
Digestive disease	325 (6.0%)	209 (6.1%)	116 (5.8%)
Perinatal condition	293 (5.4%)	184 (5.4%)	109 (5.5%)
Other condition not classified	285 (5.3%)	181 (5.3%)	104 (5.2%)
Skin condition	216 (4.0%)	147 (4.3%)	69 (3.5%)
Genitourinary disease	141 (2.6%)	82 (2.4%)	59 (3.0%)
Disease of circulatory system or blood	101 (1.9%)	46 (1.4%)	55 (2.8%)
Other	395 (7.3%)	251 (7.4%)	144 (7.2%)
**Distance from home to hospital (km)**			
Median (IQR)	3.56 (1.81; 4.82)	4.05 (2.5; 5.08)	2.58 (1.42; 4.19)
Range	0.33–13.59	0.33–13.59	0.33–8.12

Data are *n* (%) unless otherwise indicated.

ED, emergency department.

The average utilisation rate for the whole cohort was 106.12 visits per 1,000 children per year ([5,395/10,168] ÷ 5 × 1,000), and the rate was higher for children of migrant mothers as compared to children of UK/Irish-born mothers (Table 2). The results in Table 2 show that in sub-group analysis, when not adjusting for covariates, the highest rates of ED utilisation were for children whose mothers were from South Asia (112.32 visits per 1,000 children per year), from Europe or Central Asia (111.18 visits per 1,000 children per year), or considered established migrants (115.95 visits per 1,000 children per year).

Among ED users (those who used the ED at least once), the crude utilisation rates show that the highest rates of ED utilisation were by children of mothers from Europe or Central Asia (453.16 visits per 1,000 children per year) and Africa (420.83 visits per 1,000 children per year) and those most recently arrived in the UK (382.76 visits per 1,000 children per year).

### Multivariable analyses: Likelihood of any ED use

The multiple logistic regression analyses confirmed that children of migrant mothers were significantly less likely than children of UK/Irish-born mothers to have visited the ED in the first 5 years of life when adjusting for other important covariates of interest (odds ratio [OR] 0.88 [95% CI 0.80 to 0.97], *p =* 0.012) (Table 4). The findings highlight that, while children of migrant mothers from all regions appear less likely to use the ED than children of UK/Irish-born mothers, this difference is only significant for children of mothers from Europe/Central Asia (OR 0.73 [95% CI 0.55 to 0.95], *p =* 0.018) and from Africa (OR 0.68 [95% CI 0.49 to 0.95], *p =* 0.022).

**Table 4:** Unadjusted and adjusted ORs and IRRs for ED utilisation.

Model and sub-group	Likelihood of ED utilisation (odds of use) (*N* = 10,168)				Frequency of ED utilisation among ever users (*N* = 3,104)			
	Unadjusted OR (95% CI)	*p*-Value*	Adjusted OR (95% CI)	*p*-Value^	Unadjusted IRR (95% CI)	*p*-Value*	Adjusted IRR (95% CI)	*p*-Value^†^
**Model 1: Mother’s migrant status**								
*UK/Irish-born (ref)*	1.00		1.00		1.00		1.00	
Foreign-born	0.92 (0.83 to 1.00)	**0.053**	0.88 (0.80 to 0.97)	**0.012**	1.36 (1.17 to 1.58)	**<0.001**	1.19 (1.01 to 1.40)	**0.040**
**Model 2: Mother’s region of origin**								
*UK/Irish-born (ref)*	1.00		1.00		1.00		1.00	
East Asia/Pacific	0.77 (0.50 to 1.18)	0.231	0.84 (0.54 to 1.28)	0.414	1.04 (0.48 to 2.22)	0.926	1.15 (0.52 to 2.55)	0.722
Europe/Central Asia	0.72 (0.55 to 0.93)	**0.012**	0.73 (0.55 to 0.95)	**0.018**	2.09 (1.33 to 3.28)	**0.001**	1.71 (1.07 to 2.71)	**0.024**
Middle East	0.59 (0.32 to 1.08)	0.087	0.61 (0.33 to 1.13)	0.115	1.31 (0.43 to 3.99)	0.635	1.69 (0.52 to 5.46)	0.382
South Asia	0.98 (0.88 to 1.07)	0.607	0.93 (0.84 to 1.03)	0.153	1.28 (1.09 to 1.51)	**0.002**	1.11 (0.93 to 1.32)	0.257
Africa	0.65 (0.45 to 0.90)	**0.010**	0.68 (0.49 to 0.95)	**0.022**	1.78 (1.00 to 3.17)	**0.049**	1.67 (0.91 to 3.06)	0.097
**Model 3: Time since mother’s arrival in UK**								
*UK/Irish-born (ref)*	1.00		1.00		1.00		1.00	
Short term (<1 year in UK)	0.79 (0.58 to 1.06)	0.119	0.76 (0.56 to 1.03)	0.080	1.43 (0.84 to 2.43)	0.186	1.22 (0.70 to 2.11)	0.482
Long term (≥1 to <5 years in UK)	0.82 (0.71 to 0.93)	**0.003**	0.78 (0.68 to 0.90)	**0.001**	1.37 (1.08 to 1.73)	**0.009**	1.09 (0.85 to 1.39)	0.474
Established (≥5 years in UK)	0.99 (0.89 to 1.10)	0.913	0.97 (0.87 to 1.09)	0.639	1.35 (1.12 to 1.61)	**0.001**	1.24 (1.02 to 1.51)	**0.032**

Significant *p*-values (*p* < 0.05) are bolded.

*Univariable logistic regression.

^^^Multiple logistic regression.

^†^Zero-truncated negative binomial regression.

ED, emergency department; IRR, incidence rate ratio; OR, odds ratio.

Children of migrant mothers, irrespective of the mother’s time since arrival in the UK, were less likely to have visited the ED. However, the multivariable analyses illustrate that, with increasing time since the mother’s arrival in the UK, children of migrant mothers show increasingly similar patterns of utilisation compared to children of UK/Irish-born mothers.

### Models adjusted for child’s sex, distance from home to hospital, and mother’s age, parity, education, and level of deprivation (IMD quintile): Frequency of ED use

For children who had ever used the ED (*N* = 3,104), a significant association was shown between rate of ED use and migrant status when controlling for covariates of interest (IRR 1.19 [95% CI 1.01 to 1.40], *p* = 0.040) (Table 4). This indicates a higher rate of ED use by children of migrant mothers. However, this higher rate of utilisation was only significant, in sub-group analysis, for children of migrant mothers from Europe or Central Asia (IRR 1.71 [95% CI 1.07 to 2.71], *p =* 0.024) and for children of established migrants (IRR 1.24 [95% CI 1.02 to 1.51], *p =* 0.032) when compared to children of UK/Irish-born mothers.

## Discussion

In this study we observed that children born to migrant mothers were less likely than those born to UK/Irish-born mothers to make a first attendance to the ED in the first 5 years of life. Children of migrant and non-migrant mothers attended the ED on similar types of days and with similar conditions, and similar proportions were admitted to hospital. However, among the sub-population of children who attended the ED, those born to migrant mothers had a higher utilisation rate compared to children of UK/Irish-born mothers. This indicates a higher rate of return to the ED for children of migrant mothers once the service has been accessed. Although the rates of use were found to be statistically significantly different, the absolute differences in ED utilisation between children of migrants and non-migrant mothers, both for first use (5.86 additional ED visits per 1,000 person-years) and for repeated use (40.99 additional ED visits per 1,000 person-years), were rather small. These findings highlight the importance of analysing both the likelihood and the volume of service utilisation separately when seeking to understand patterns of ED utilisation.

Our findings show that after adjusting for important covariates, children of migrant mothers had a lower odds of first ED use in the first 5 years of life. To our knowledge, the only other study looking at paediatric ED use by children born to migrant mothers, although conducted in a different context, found contrasting results [23]. Our findings may suggest that either (1) children of migrant mothers in Bradford are generally not an unwell population in need of emergency care or (2) children of migrant mothers did not utilise the ED because they received care elsewhere. Of more concern is the possibility that medical care was not sought when children were in need. Existing evidence suggests that migrants in more vulnerable circumstances, such as undocumented migrants, experience substantial barriers to care [24–27].

Time since mother’s arrival in the UK was found to be an important factor in understanding likelihood of ED use. No previous studies looking at paediatric ED utilisation by children of migrant mothers have accounted for this as an explanatory factor. The odds of ED use were lowest for children of short-term migrants, and, with increasing length of stay in the UK, the difference in likelihood of first use for children of migrant and non-migrant mothers was seen to diminish. These findings may demonstrate that the migrant mothers who most recently arrived in the UK may be unfamiliar with the healthcare system and may not initially seek care for their children in the ED. With increasing length of stay in the UK, migrants’ understanding of the health service may develop, and in turn their children’s likelihood of paediatric ED use becomes more similar to that of children of UK/Irish-born mothers.

Despite all sub-groups of children born to migrant mothers in the cohort being less likely to have a first visit to the ED, once ED services were accessed, migrant mothers were found to be more likely to return to the service. This finding reflects those of other studies [23] and has important service implications for EDs and the wider healthcare system. Frequent paediatric ED visits may be expected if this population has higher levels of underlying chronic illness [28]. However, frequent attendances may also reflect access barriers migrants face when seeking other forms of healthcare [23,29], poor understanding of the host country’s healthcare system, prior positive experiences of the ED, and previous experiences within the healthcare system that result in a preference for seeking care in the ED [29–32]. Parents may also access the ED because this model of care most closely resembles the healthcare service in their home country.

ED utilisation rates were higher, but variable, for children of migrant mothers from all regions as compared to children of UK/Irish-born mothers. In particular, the results show significantly higher rates of utilisation by children of mothers from Europe/Central Asia and from Africa. Higher rates of ED utilisation by migrants by region of origin have been found in other studies [23,33–36]. Recognising the heterogeneity within the migrant population, and identifying differential use by people from different nation states and ethnicities, is important to enable health services to better understand population healthcare needs and to target health policies and interventions to meet these needs. Although, in our study, the effect sizes for some migrant populations did not reach statistical significance and have wide confidence intervals, most likely due the size of the sub-group, the patterns of utilisation by these sub-groups may have clinical and public health relevance and should be explored further.

Children of migrant mothers, irrespective of time since the mother’s arrival in the UK, showed higher frequency of ED utilisation following first access. When adjusted for covariates, ED utilisation rates remained significantly higher for children of established migrants. This contradicts the expectation that, over time, as migrant mothers become more familiar with the healthcare services in the UK, their rate of paediatric ED utilisation will become more similar to that of UK/Irish-born mothers. One possible explanation for these findings is that some children in this cohort born to established migrant mothers may have greater or more complex healthcare needs. This may be a reasonable explanation given that in Bradford the infant mortality rate is higher than the national average, along with high levels of morbidity within the Bradford population [14]. A lack of data meant that health status could not be controlled for. Other explanations again may include a range of demand- and supply-side factors including satisfaction with previous ED encounters, barriers to accessing out-of-hours services, the convenience of out-of-hours services, and long waiting times for primary care appointments [30,37].

This study has several limitations. A lack of clinical information made it impossible to analyse the acuity with which children presented, and children’s underlying morbidity may confound these results. The high proportion of hospital admissions for children of migrant mothers does suggest that for many of these children the utilisation of the ED was for conditions that require ongoing medical care. Therefore, one possible explanation for the higher rate of ED utilisation among children of migrant mothers may be that a child’s underlying health status drives both first ED use and subsequent ED use. If migrant mothers with the sickest children make use of the ED, it would not be unexpected that these children, due to the severity of their condition, are more likely to visit the ED more frequently.

A further limitation to this study is sources of selection bias in the BiB cohort, as well as bias that may have arisen from restricting the analytic cohort to mothers for whom there were complete data on the country of birth and other covariates of interest. Although the participation rate for the BiB study was not determined for migrant as compared to non-migrant mothers, some small differences overall between women recruited and those not recruited are evident in the BiB cohort [14]. Women who were recruited were older and lived in less deprived circumstances. In addition, more South Asian women than women from other regions were recruited to the BiB cohort. Thus, selection bias may be present in this study if migrants were less likely to join the BiB cohort, as might be the case particularly for vulnerable migrants such as those living in the most deprived areas and those most recently arrived in the UK. A further source of potential bias is evident in this study: A larger percentage of migrant mothers and less educated mothers were excluded from the analytical cohort due to missing data (S1 Table). These 2 sources of bias may have selected educated migrant mothers with higher socio-economic status and better language skills into the study, who would be expected to differ less from UK/Irish-born mothers. Thus, the findings of this study might have underestimated migrants’ use of services, or underestimated the use of ED services by some migrant populations.

It is possible that ED attendances for children of both migrant and non-migrant mothers were underestimated if children were taken to EDs outside of Bradford when in need of urgent care. Migrant populations are relatively mobile and may have been more likely to have sought healthcare in other EDs. Although BRI is the only ED facility within Bradford, and for 98.6% of children in the cohort was the closest ED to their home at the time of birth, it is possible that urgent and emergency care was sought in other EDs.

A further limitation is that due to incomplete data, we were unable to account for multiple children per mother within the cohort. However, by adjusting for mother’s parity, the effect of having additional children, and the experience that this brings, will have been adjusted for. Finally, the BiB cohort largely included children with mothers of British and Pakistani origin and thus may be quite different from other populations [14]. However, because we analysed the findings by maternal region of origin sub-groups, the findings may be more generalisable to other populations.

Our findings add to our knowledge and highlight differences in ED utilisation patterns between children of migrant and non-migrant mothers. The challenge for healthcare services is to identify those children accessing EDs most and to work with their parents to better understand their healthcare needs. It is important to understand when, and why, migrant and non-migrant mothers make their first ED visit for their child and, importantly, why some migrant mothers choose to return to the service.

Our study found that children of migrant mothers were less likely to use the ED for the first time in the first 5 years of life than children of non-migrant mothers, but, once accessed, the rate of repeat attendance was higher. Our findings confirm that immigrant groups use the ED differently when the analysis is adjusted for covariates of interest. Further research and better data are needed to understand these patterns of utilisation, the variations in use between people with different origins and backgrounds, and the reasons for these differences in utilisation. Understanding the reasons for frequent or repeated ED use among some migrant groups is important to ensure that the healthcare service is meeting the needs of the demographically changing population, while simultaneously addressing demand in paediatric EDs.

## Supporting information

S1 Checklist Strengthening the reporting of observational studies in epidemiology (STROBE) checklist. (DOCX)

S1 Table Comparison of analytic cohort and cohort excluded from analysis due to missing data. (DOCX)

## Abbreviations

BiB: Born in Bradford
BRI: Bradford Royal Infirmary
ED: emergency department
GP: general practitioner
IMD: Index of Multiple Deprivation
IRR: incidence rate ratio
NHS: National Health Service
OR: odds ratio
