# Supplementary material for: Paediatric emergency department utilisation rates and maternal migration status in the Born in Bradford cohort: A cross-sectional study
Source: PLoS Med. 2020 Mar 3;17(3):e1003043. doi: 10.1371/journal.pmed.1003043 (PMC7053707; doi:10.1371/journal.pmed.1003043)
Supplement: S1 Table — (DOCX) [file pmed.1003043.s002.docx]

|  | INCLUDED IN ANALYSIS  N= 10,168 | EXCLUDED FROM ANALYSIS^1^  N= 3,264 | p-value^$,*^ |
| --- | --- | --- | --- |
|  | **N (%)** | **N (%)** |  |
| N.of ED visits (first 5 years)  No visits  At least one visit | 7,064 (69.5%)  3,104 (30.5%) | 2,260 (69.2%)  1,004 (30.8%) | 0.16; p=0.685^$^ |
| Mother’s migrant status  UK/Ireland born  Migrant  Missing | 6,548 (64.4%)  3,620 (35.6%)  - | 484 (51.8%)  451 (48.2%)  *2,329* | 58,8 (1); p<0.001^$^ |
| Child gender  Male  Female | 5,157 (50.7%)  5,011 (49.3%) | 1,696 (52.0%)  1,568 (48.04%) | 1.5 (1); p=0.217^$^ |
| Mother’s age at recruitment  Med (IQR) | 27 (23; 31) | 27 (23; 31) | Z=1.181; p=0.238* |
| Registerable parity  No previous birth  At least one previous birth  Missing | 4,179 (41.1%)  5,989 (58.9%)  - | 828 (33.5%)  1,640 (66.5%)  *796* | 47.32; p<0.001^$^ |
| Mother educated beyond A level or equivalent  < A level or equivalent  A-level equivalent or higher  Don’t know or foreign unknown  Missing | 5,286 (52.0%)  4,670 (45.9%)  212 (2.1%)  - | 477 (52.7%)  406 (44.9%)  22 (2.4%)  *2,359* | 114.18; p<0.001^$^ |
| Marital and cohabitation status  Married and living with partner  Not married, living with partner  Not living with partner  Missing | 6,642 (65.3%)  1,838 (18.1%)  1,688 (16.6%)  - | 621 (68.62%)  143 (15.80%)  141 (15.6%)  *2,359* | 4.31; p=0.116^$^ |
| Residential deprivation IMD quintile 2010  1 (most deprived)  2  3  4  5 (least deprived)  Missing | 6,712 (66.0%)  1,838 (18.1%)  1,141 (11.2%)  302 (3.0%)  175 (1.7%)  - | 635 (68.7%)  172 (18.6%)  77 (8.3%)  28 (3.0%)  12 (1.4%)  *2,340* | 8.41; p=0.077^$^ |

**S1 Table: Comparison of analytic cohort and cohort excluded from analysis due to missing data.**

^1^Distribution of those excluded from analytical cohort due to missing data (N=3,264) by available outcome, exposure and covariate data.

IQR: interquartile range

^$^ Chi-square analysis

*Mann-Whitney U-test
